# ATM Limits Incorrect End Utilization during Non-Homologous End Joining of Multiple Chromosome Breaks

**DOI:** 10.1371/journal.pgen.1001194

**Published:** 2010-11-04

**Authors:** Nicole Bennardo, Jeremy M. Stark

**Affiliations:** Department of Cancer Biology and Irell and Manella Graduate School of Biological Sciences, Beckman Research Institute of the City of Hope, Duarte, California, United States of America; University of Washington, United States of America

## Abstract

Chromosome rearrangements can form when incorrect ends are matched during end joining (EJ) repair of multiple chromosomal double-strand breaks (DSBs). We tested whether the ATM kinase limits chromosome rearrangements via suppressing incorrect end utilization during EJ repair of multiple DSBs. For this, we developed a system for monitoring EJ of two tandem DSBs that can be repaired using correct ends (Proximal-EJ) or incorrect ends (Distal-EJ, which causes loss of the DNA between the DSBs). In this system, two DSBs are induced in a chromosomal reporter by the meganuclease I-SceI. These DSBs are processed into non-cohesive ends by the exonuclease Trex2, which leads to the formation of I-SceI–resistant EJ products during both Proximal-EJ and Distal-EJ. Using this method, we find that genetic or chemical disruption of ATM causes a substantial increase in Distal-EJ, but not Proximal-EJ. We also find that the increase in Distal-EJ caused by ATM disruption is dependent on classical non-homologous end joining (c-NHEJ) factors, specifically DNA-PKcs, Xrcc4, and XLF. We present evidence that Nbs1-deficiency also causes elevated Distal-EJ, but not Proximal-EJ, to a similar degree as ATM-deficiency. In addition, to evaluate the roles of these factors on end processing, we examined Distal-EJ repair junctions. We found that ATM and Xrcc4 limit the length of deletions, whereas Nbs1 and DNA-PKcs promote short deletions. Thus, the regulation of end processing appears distinct from that of end utilization. In summary, we suggest that ATM is important to limit incorrect end utilization during c-NHEJ.

## Author Summary

When a chromosome is fragmented by multiple double-strand breaks (DSBs), each set of DSB ends needs to be matched correctly during repair to avoid chromosomal rearrangements. Considering the case of two tandem DSBs, if the ends of different breaks (incorrect ends) are used for repair, loss of the intervening DNA can occur. Alternatively, when the ends of a single DSB (correct ends) are used for repair, the original order of the chromosome is restored. Deficiencies in the factors ATM and Nbs1, as seen in patients with Ataxia Telangiectasia and Nijmegen Breakage Syndrome, respectively, have been associated with elevated chromosome rearrangements and cancer predisposition. Hence, we examined the possibility that these factors may be important for the usage of correct ends during repair of multiple DSBs. For this, we developed a reporter system to examine end usage during repair of two tandem DSBs in mammalian chromosomes and found that disruption of ATM or Nbs1 leads to elevated usage of incorrect ends. Furthermore, we found that the role of ATM during end usage depends on a repair pathway called classical non-homologous end joining (c-NHEJ). We suggest that ATM suppresses genome rearrangements via limiting incorrect end utilization during c-NHEJ.

## Introduction

Recent sequencing of cancer genomes has revealed a prevalence of chromosome rearrangements, including interchromosomal translocations and intrachromosomal rearrangements [1]. These rearrangements could arise from end joining (EJ) of incorrect ends of multiple chromosomal double-strand breaks (DSBs). Such EJ could be performed by classical non-homologous end joining (c-NHEJ) factors that mediate V(D)J Recombination (e.g. Ku70/Ku80, XLF, DNA-PKcs, and Xrcc4/Lig4), or by Alternative-EJ (alt-EJ) pathways that are independent of these factors [2], [3]. We suggest that identifying the mechanisms that are important for the fidelity of end utilization during c-NHEJ and/or alt-EJ will provide insight into maintenance of chromosome stability and tumor suppression.

Factors that reduce incorrect end utilization during EJ are likely to be important for suppressing chromosome rearrangements. Mutations in the ATM kinase, found in patients with the genetic disorder Ataxia-Telangiectasia (A-T), cause elevated levels of chromosomal abnormalities, along with a predisposition for cancer [4]. Part of the role of ATM in suppressing chromosomal abnormalities is likely related to its key function during the DNA Damage Response (DDR). Without the DDR, cells fail to activate cell cycle checkpoints following DNA damage, and are more likely to undergo DNA replication and/or mitosis with broken chromosomes, which could lead to rearrangements [5]. Also important for the DDR is Nbs1, which is a member of the Mre11-complex (Mre11-Rad50-Nbs1) and is important for ATM activation [6], [7]. Patients with mutations in the *Nbs1* gene (Nijmegen Breakage Syndrome), like A-T patients, show cancer predisposition associated with elevated chromosomal abnormalities [8].

ATM and Nbs1 localize to sites of DSBs, and are important for their repair [6], [9]. Both ATM and Nbs1 are important for cell survival following ionizing radiation (IR)-induced DSBs [4], [8], and promote homologous recombination [10]. Also consistent with a role in repair, a subset of IR-induced DSBs persist in ATM-deficient cells [11]. Persistent breaks have also been observed in ATM-deficient lymphocytes during V(D)J recombination and class switch recombination (CSR) [12]–[15]. ATM and Nbs1 affect the repair fidelity of certain V(D)J recombination substrates, in which the Rag1/2 nuclease forms two types of DSB ends: hairpin coding-ends and blunt signal-ends [13], [16], [17]. Correct end utilization in this context involves pairing coding-coding and signal-signal ends during NHEJ. When Rag1/2 cleavage sites are placed in an inverted orientation, both ATM and Nbs1 have been shown to suppress hybrid signal-coding EJ products [13], [16]–[18]. Thus, these factors are important for faithful repair of Rag1/2-induced DSBs. Consistent with this notion, ATM-deficient lymphocytes show elevated chromosome rearrangements resulting from V(D)J recombination [19], [20]. ATM and Nbs1 also promote efficient CSR and suppress translocations between *IgH* and *c-myc* during this process [9], [12], [21]–[25]. Similarly, the ATM orthologue in yeast (*TEL1*) is important to suppress translocations in favor of intrachromosomal EJ [26].

Thus, we considered the possibility that ATM and/or Nbs1 play a role in correct end utilization during EJ repair of multiple chromosomal DSBs in mammalian cells, outside of the programmed rearrangements during lymphocyte development. For this, we monitored EJ products following the induction of two tandem DSBs, which can be repaired using either correct ends (Proximal-EJ) or incorrect ends (Distal-EJ). We find that disruption of ATM or Nbs1 causes elevated Distal-EJ, but not Proximal-EJ. Furthermore, the elevation of Distal-EJ caused by ATM-disruption is dependent on the c-NHEJ factors DNA-PKcs, Xrcc4, and XLF. In addition, to examine the role of these factors on end processing, we analyzed Distal-EJ repair junctions. We find that ATM and Xrcc4 limit extensive deletions during EJ, whereas Nbs1 and DNA-PKcs promote short deletions. Thus, the role of individual factors during end processing does not directly correlate with their roles during end utilization. In summary, we suggest that ATM is important to limit incorrect end utilization during repair by c-NHEJ.

## Results

### Reporter for Distal-EJ versus Proximal-EJ of two tandem DSBs

**Figure 1:**
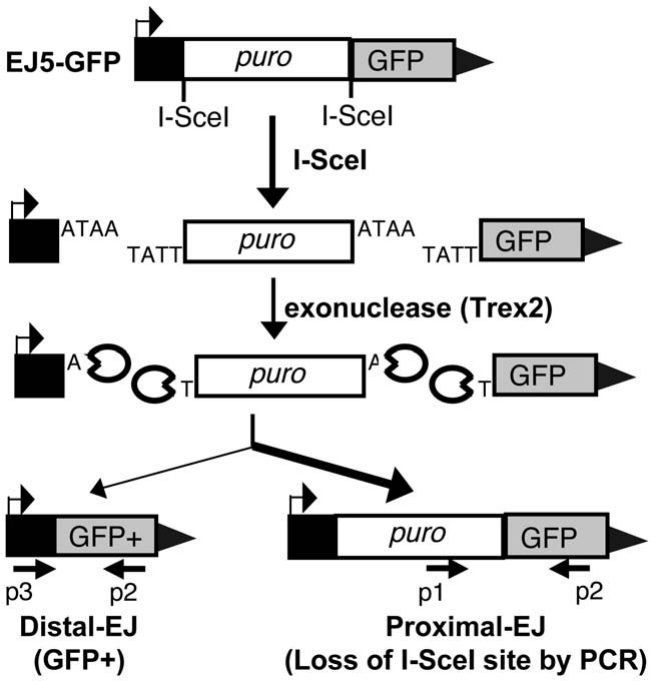
Measuring end utilization during EJ repair of two tandem DSBs. Shown is the EJ5-GFP reporter, which contains a GFP coding sequence that is separated from its promoter by a puromycin resistance gene (*puro*) that is flanked by two tandem I-SceI sites. We express I-SceI to induce two DSBs with four nt. 3′ overhangs, and co-express Trex2 to cause partial degradation of these overhangs, leading to I-SceI-resistant EJ products. Shown on the left is the Distal-EJ repair product that causes the deletion of *puro* and restoration of the *GFP+* cassette, which can be measured by FACS analysis. Also depicted are two primers (p3, p2) for analysis of Distal-EJ repair junctions. Shown on the right is the Proximal-EJ product, which is quantified by I-SceI digestion analysis of an amplification product that spans the 3′ I-SceI site (primers p1, p2). This approach enables quantification of two different I-SceI-resistant EJ products from the same sample: Distal-EJ (%GFP+ cells) and Proximal-EJ (% I-SceI-resistant product). Proximal-EJ is much more efficient than Distal-EJ in WT cells, as represented by the heavier arrow.

We investigated incorrect and correct end utilization during EJ repair of two tandem DSBs. In this context, incorrect end utilization involves the joining of distal DSB ends (Distal-EJ), as this repair event leads to an intrachromosomal deletion between the two DSBs. In contrast, correct end utilization maintains proximal ends during repair (Proximal-EJ). We developed a method to measure Proximal-EJ and Distal-EJ repair of two tandem chromosome breaks generated by the meganuclease I-SceI, using the reporter EJ5-GFP (Figure 1) [27], [28]. In this reporter, a promoter is separated from the rest of a GFP expression cassette by 1.7 kb (*puro* cassette) that is flanked by two tandem I-SceI sites. Following I-SceI expression, Distal-EJ places the promoter adjacent to the rest of the GFP-expression cassette, such that Distal-EJ can be quantified as the percentage of GFP+ cells [27], [28].

Proximal-EJ is difficult to measure with I-SceI expression alone, since EJ that restores the I-SceI site cannot be differentiated from the uncut reporter [27]. Thus, we adapted this reporter system to enable the quantification of I-SceI-resistant Proximal-EJ products by co-expressing I-SceI with a non-processive 3′ exonuclease (Trex2). As described previously, expression of Trex2 appears to cause partial degradation of the 4 nt. 3′ cohesive ends generated by I-SceI, such that co-expression of I-SceI with Trex2 leads to a high level of I-SceI-resistant Proximal-EJ products [27]. Thus, Proximal-EJ can be quantified by loss of the I-SceI site through PCR amplification across the 3′ I-SceI site, and subsequent I-SceI digestion analysis (Figure 1, primers p1, p2). In this assay we determine the percentage of I-SceI-resistant events by quantifying the relative intensity of the I-SceI-resistant and I-SceI-sensitive products within the same sample. This approach has been described previously for other I-SceI assays [29], and confirmed here to be quantitative within at least two-fold (Figure S1A).

**Figure 2:**
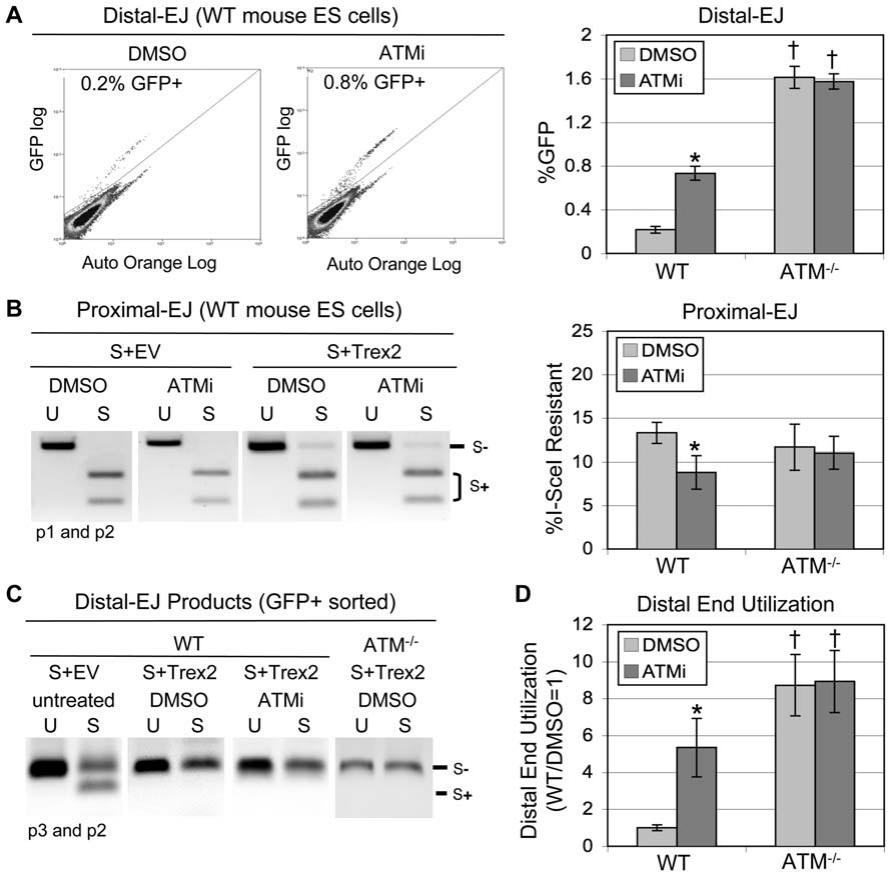
ATM suppresses Distal End Utilization. EJ5-GFP was integrated into wild type (WT) and *ATM^−/−^* mouse ES cells. These lines were co-transfected with expression plasmids for I-SceI and Trex2, and treated with ATMi or DMSO (vehicle). Subsequently, frequencies of Distal-EJ and Proximal-EJ were determined as described in Figure 1. A. ATM suppresses Distal-EJ. Shown (left) are two representative FACS profiles for WT mouse ES cells transfected and treated with DMSO or ATMi, as described above. Also shown (right) are the mean GFP+ frequencies (Distal-EJ) for WT and *ATM^−/−^*cells transfected and treated with DMSO or ATMi, as described above (N = 6, error bars denote s.d.). (*) statistical difference between DMSO and ATMi treatments of the same cell line (p<0.0001), (†) statistical difference between WT and mutant ES cell lines under the same treatment conditions (p<0.0001). B. Proximal-EJ is modestly reduced by ATMi treatment of WT cells, but is the same for WT and *ATM^−/−^* cells. Formation of an I-SceI-resistant EJ product at the 3′ I-SceI site (Proximal-EJ) was determined by amplification (primers p1, p2) of genomic DNA samples from the transfection experiments shown in A, followed by I-SceI digestion analysis. Shown (left) are representative samples of uncut (U) and I-SceI-digested (S) products following co-transfection of expression vectors for I-SceI and Trex2 (S+Trex2), or co-transfection of an expression vector for I-SceI with empty vector (S+EV). Also shown (right) are the mean Proximal-EJ frequencies from the identical transfection samples shown in A (N = 6, error bars denote s.d.). (*) as in A (p = 0.0007). C. Co-expression of I-SceI and Trex2 leads to Distal-EJ products that are I-SceI-resistant. The Distal-EJ junctions were amplified from genomic DNA of sorted GFP+ cells (primers p2, p3) from a representative of each transfection described in A. Shown are uncut (U) and I-SceI-digested (S) amplification products from these samples. D. Disruption of ATM leads to elevated Distal End Utilization. Distal End Utilization was calculated by dividing Distal-EJ (% GFP+ cells) by Proximal-EJ (% I-SceI resistant p1, p2 product) for individual samples of the transfections described in A. Mean values are depicted relative to the mean Distal End Utilization value for WT DMSO-treated cells (N = 6, error bars denote s.d.). (*) as in A (p<0.0001). (†) as in A (p≤0.0035).

Using this assay system, Proximal-EJ has been shown to be substantially more efficient than Distal-EJ [27]. To confirm this finding, we co-expressed I-SceI with Trex2 in a WT mouse ES cell line with a chromosomally integrated copy of EJ5-GFP [28], and analyzed the EJ repair products, as described above (Figure 1). From these experiments, we observed low levels of Distal-EJ (0.2% GFP+ cells, Figure 2A), and much higher levels of Proximal-EJ (13% I-SceI-resistant p1,p2 amplification products, Figure 2B). In contrast, following transfection of I-SceI without Trex2, we found no detectable I-SceI-resistant Proximal-EJ products (Figure 2B). These results indicate that Proximal-EJ predominates following Trex2 and I-SceI co-expression, such that this experimental approach may uncover factors important for correct end utilization that are otherwise masked from experiments using I-SceI expression alone.

Importantly, the Distal-EJ products resulting from co-expression of I-SceI and Trex2 are also completely I-SceI-resistant [27]. We have confirmed this notion here, using GFP+ sorted samples from the aforementioned transfection experiment (Figure 2C, Figure S1B). Therefore, this method can be used to measure the frequency of two different I-SceI-resistant products (Distal-EJ and Proximal-EJ) from a single sample (Figure 1).

### ATM-disruption causes elevated Distal-EJ but not Proximal-EJ

We considered the possibility that ATM may affect end utilization during EJ, as this factor is important for chromosome stability [4]. To test this hypothesis, EJ5-GFP was chromosomally integrated into *ATM^−/−^* mouse ES cells [30], and analyzed in parallel with the WT ES cell line described above. Furthermore, during co-expression of I-SceI and Trex2, we treated the cells with either a highly-specific ATM kinase inhibitor (ATMi) [31] or vehicle (DMSO). Subsequently, the percentage of GFP+ cells (Distal-EJ) was measured by FACS analysis. From these experiments, we found that ATMi treatment of WT cells caused an increase in Distal-EJ, as compared to DMSO treated cells (3.4-fold, p<0.0001, Figure 2A). Similarly, *ATM^−/−^* cells exhibited higher levels of Distal-EJ as compared to WT cells (7.4-fold, p<0.0001, Figure 2A). Finally, treatment of the *ATM^−/−^* cells with ATMi had no effect on Distal-EJ, which is consistent with the high-specificity of ATMi [31]. These data indicate that ATM kinase activity is important for the suppression of Distal-EJ.

To measure Proximal-EJ, we isolated genomic DNA from the same samples used in the FACS analysis, and determined the percentage of I-SceI-resistant amplification products, as described above (Figure 1, primers p1, p2). For both WT and ATM-deficient cells, we found that co-expression of I-SceI and Trex2 results in a significant level of I-SceI-resistant Proximal-EJ products, which are not detectable from expression of I-SceI alone (S+Trex2 versus S+EV, respectively, Figure 2B, Figure S1C). Regarding frequencies, we found that WT and *ATM^−/−^* cells exhibited equivalent levels of Proximal-EJ (Figure 2B). ATMi treatment caused a modest reduction in Proximal-EJ in WT cells (Figure 2B, 1.5-fold, p = 0.0007), but not in *ATM^−/−^* cells. Thus, loss of ATM kinase activity, but not complete disruption of ATM, appears to modestly reduce Proximal-EJ. Importantly, neither ATMi nor genetic disruption of *ATM* caused an increase in Proximal-EJ.

We then directly compared Distal-EJ and Proximal-EJ values to determine the relative frequency of incorrect end utilization (Distal End Utilization). First, we confirmed that the Distal-EJ products (GFP+) formed following I-SceI and Trex2 co-expression were I-SceI-resistant for all cell types. We sorted GFP+ cells, isolated genomic DNA, amplified the Distal-EJ products (Figure 1, primers p2, p3), and performed I-SceI digestion analysis. We found that Distal-EJ products were completely I-SceI-resistant for both WT and ATM-deficient cells following I-SceI and Trex2 co-expression, unlike Distal-EJ products resulting from transfection with I-SceI alone (Figure 2C, Figure S1B). We then quantified Distal End Utilization by calculating the ratio of Distal-EJ versus Proximal-EJ for individual samples. From this analysis, we found that ATMi-treatment of WT cells led to a substantial increase in Distal End Utilization in comparison to DMSO treated cells (Figure 2D, 5.3-fold, p<0.0001). Similarly, *ATM^−/−^* cells exhibited a striking increase in Distal End Utilization, in comparison to WT cells (Figure 2D, 8.7-fold, p<0.0001). Last, ATMi-treatment of *ATM^−/−^* cells did not affect Distal End Utilization.

In the above experiments, ATM appears to suppress Distal-EJ without promoting Proximal-EJ to a similar degree. However, in all conditions, Proximal-EJ is predominant (e.g. 13% for WT, 11.7% for *ATM^−/−^*) over the minor Distal-EJ product (e.g. 0.2% for WT, 1.6% for *ATM^−/−^*). Thus, Distal-EJ is relatively infrequent, as compared to Proximal-EJ. Accordingly, the fold-increase in Distal-EJ caused by ATM-disruption would not necessarily be matched by a similar fold-decrease in Proximal-EJ. Considering one other detail of these experiments, we note that determining the effect of ATMi on Distal End Utilization after 6 days of culturing post-transfection was not statistically different from the 3 days protocol described in Figure 2 (Figure S1D). This finding indicates that 3 days is a reasonable end-point for these experiments.

We next considered the possibility that ATM might inhibit the formation of both I-SceI-induced DSBs, which would limit Distal-EJ. For this, we used clonal analysis to determine the frequency of I-SceI-resistant Proximal-EJ products at both tandem I-SceI sites. Specifically, we expressed I-SceI and Trex2 in WT ES cells treated with ATMi or DMSO. Following the usual 3 days of culturing, we plated transfected cells at low density to isolate single clones. For individual clones, we determined whether the 5′ and/or 3′ I-SceI recognition sites had been lost, by performing PCR amplification and I-SceI digestion analysis. From this experiment, we found that clones with loss of either the 5′ or 3′ I-SceI site frequently lost the second site (Figure S2A; WT DMSO treated cells: 19 clones lost both 5′ and 3′ sites, 18 clones lost only one of the sites). Thus, cutting at two tandem I-SceI sites, followed by EJ that leads to I-SceI-resistant products at both sites, appears efficient in WT cells. Furthermore, ATMi treatment did not cause an increase in clones that lost both I-SceI sites (Figure S2A; WT ATMi treated cells: 9 clones lost both 5′ and 3′ sites, 25 clones lost only one of the sites). These results indicate that ATM does not suppress the formation of tandem I-SceI-induced DSBs. However, we note that these experiments do not address potential effects of ATM on the probability that both DSBs persist simultaneously (see break persistence model in Discussion).

We also performed the ATMi analysis in a distinct cell type: a transformed human embryonic kidney cell line (HEK293, Figure S2B) that contains a chromosomally integrated EJ5-GFP reporter [28]. Using this HEK293-EJ5-GFP cell line for the same transfection experiment described for ES cells, we found that ATMi treatment caused elevated levels of Distal-EJ (2.1-fold, p<0.0001), but did not affect Proximal-EJ, leading to an increase in Distal End Utilization (Figure S2B, 2.2-fold, p<0.0001). Combined, these findings indicate that ATM is important for limiting Distal End Utilization during EJ repair of multiple DSBs in both mouse ES and human HEK293 cells.

### ATM limits Distal-EJ only in c-NHEJ proficient cells

**Figure 3:**
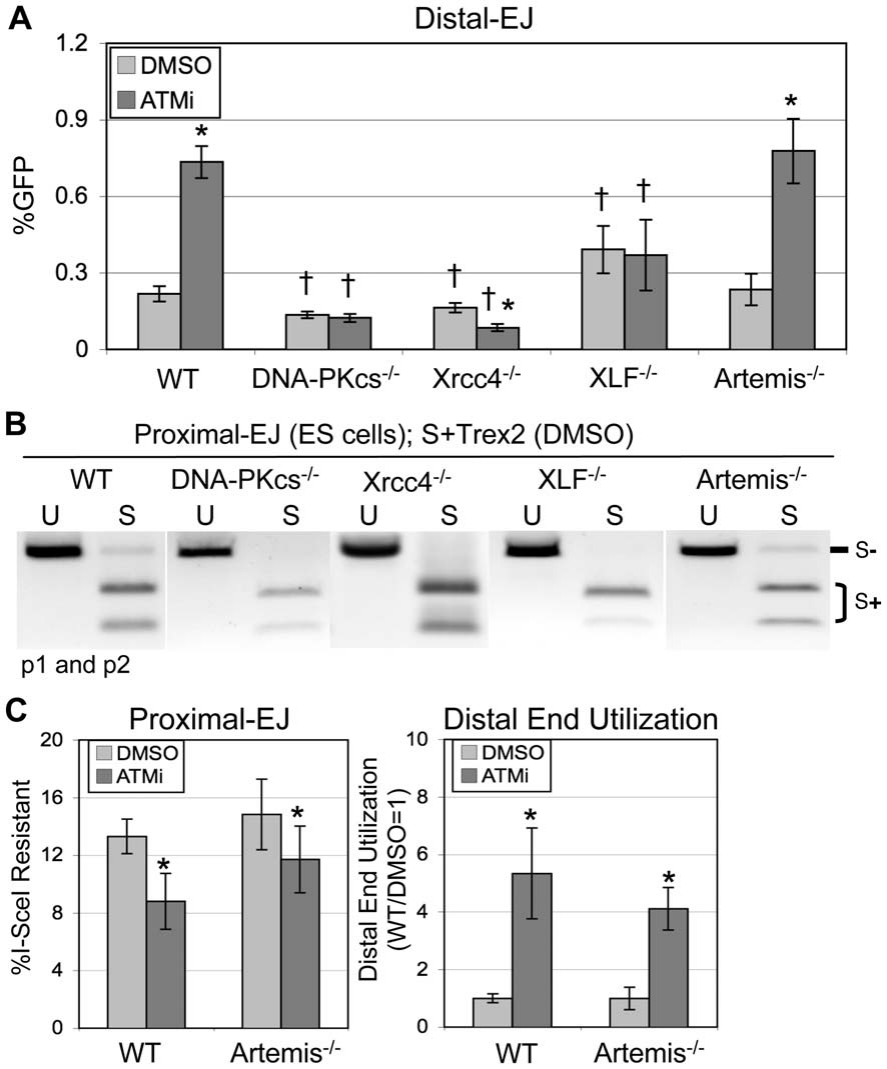
The elevation in Distal-EJ caused by ATMi is dependent on DNA-PKcs, Xrcc4, and XLF, but not Artemis. A. Shown are mean Distal-EJ frequencies for WT, *DNA-PKcs^−/−^*, *Xrcc4^−/−^*, *XLF^−/−^*, and *Artemis^−/−^* mouse ES cell lines, each with an integrated EJ5-GFP reporter, which were transfected and treated with DMSO or ATMi, as described in Figure 2 (N = 6, error bars denote s.d.). (*) statistical difference between DMSO versus ATMi treatments of the same cell line (p<0.0001), (†) statistical difference between WT and mutant ES cell lines of the same treatment (p≤0.0014). B. Proximal-EJ requires DNA-PKcs, Xrcc4, and XLF, but not Artemis. Shown are representative samples of uncut (U) and I-SceI-digested (S) p1, p2 amplification products from representative transfections described in A. C. The effect of ATMi on EJ is not distinct between WT and *Artemis^−/−^* cells. Shown (left) are the mean frequencies of Proximal-EJ for the WT and *Artemis^−/−^* transfection experiments described in A (N = 6, error bars denote s.d.). (*) as in A, p<0.0001 for WT, p = 0.0461 for *Artemis^−/−^*. Shown (right) are mean Distal End Utilization values of individual samples relative to DMSO-treated WT cells (N = 6, error bars denote s.d.). (*) as in A (p<0.0001).

We next tested whether the increase in Distal-EJ that is caused by ATM-disruption involves c-NHEJ factors, specifically DNA-PKcs, Xrcc4, and/or XLF. DNA-PKcs is recruited to DSBs by the Ku70/Ku80 heterodimer, and can stabilize the two ends of a DSB prior to ligation by the Xrcc4/Lig4 complex, which is promoted by XLF [2]. We integrated EJ5-GFP into *DNA-PKcs^−/−^* [32], *Xrcc4^−/−^* [33], and *XLF^−/−^* [34] ES cells, and analyzed EJ efficiency in these cell lines following expression of I-SceI and Trex2, along with ATMi or DMSO treatment, as described above for WT cells. From these experiments (Figure 3A), we found that ATMi treatment did not affect Distal-EJ in *DNA-PKcs^−/−^* and *XLF^−/−^* cells, and caused a decrease in Distal-EJ in *Xrcc4^−/−^* cells (1.9-fold, p<0.0001), all of which are distinct from the 3.4-fold increase observed in WT cells. Importantly, these results indicate that DNA-PKcs, Xrcc4, and XLF are essential for the increase in Distal-EJ caused by ATM-disruption.

Regarding overall frequencies of EJ in ATM-proficient cells, we observed some differences between WT versus *DNA-PKcs^−/−^*, *Xrcc4^−/−^*, and *XLF^−/−^* cells. For instance, we found that I-SceI-resistant Proximal-EJ products were below the level of detection for both the *DNA-PKcs^−/−^* and *XLF^−/−^* cells (<2%, Figure 3B), similar to previous findings in *Xrcc4^−/−^* cells [27] that we have repeated here (Figure 3B). These results indicate that DNA-PKcs, Xrcc4, and XLF are essential for significant levels of Proximal-EJ of DSB ends processed by Trex2. Consistent with these results, DNA-PKcs, Xrcc4/Ligase IV, and XLF were previously shown to promote NHEJ of non-cohesive DSB ends *in vitro* [35]. Regarding Distal-EJ frequencies compared to WT cells, *DNA-PKcs^−/−^* and *Xrcc4^−/−^* cells showed a reduction (1.6-fold, p<0.0001, and 1.3-fold, p = 0.0041, respectively, Figure 3A), whereas *XLF^−/−^* cells exhibited an increase in Distal-EJ (1.8-fold, p = 0.0014, Figure 3A). Unfortunately, since Proximal-EJ is below the limit of detection in the c-NHEJ-deficient cells, it is not possible to quantify Distal End Utilization for these cell lines. Nevertheless, Proximal-EJ is substantially reduced in these cells (<2%) compared to WT cells (13%), whereas Distal-EJ levels in these cells are within 2-fold of WT cells. These results indicate that DNA-PKcs, Xrcc4, and XLF are important for correct end utilization.

We then sought to determine whether the EJ events measured with EJ5-GFP may be mechanistically distinct from c-NHEJ during V(D)J recombination. For this, we examined the c-NHEJ factor Artemis: a nuclease that is important for hairpin opening during V(D)J recombination [2]. We integrated the EJ5-GFP reporter into *Artemis^−/−^* ES cells [36], and performed the transfection analysis described above. In contrast to the above c-NHEJ factors, *Artemis^−/−^* cells showed no clear distinction from WT cells on the frequencies of Distal-EJ, Proximal-EJ, or Distal End Utilization, nor on the effect of ATMi treatment on these EJ events (Figure 3). These results indicate that Artemis is not involved in these EJ processes, which provides a contrast to the findings with DNA-PKcs, Xrcc4, and XLF. These findings also confirm the notion that repair of the EJ events measured here show mechanistic distinctions from the hybrid coding-signal joints of V(D)J recombination substrates, which are also elevated in ATM-deficient cells, yet require Artemis [2], [18], [36].

### Nbs1 limits Distal-EJ but not Proximal-EJ, similar to ATM

**Figure 4:**
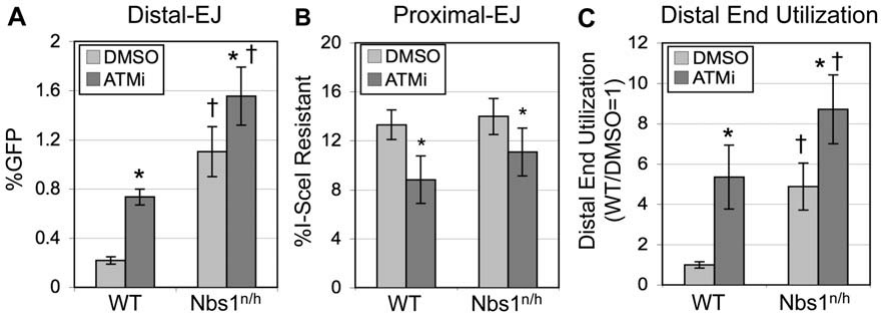
Nbs1 suppresses Distal-EJ to a similar degree as ATM. A. Distal-EJ is elevated in Nbs1-deficient cells, and ATMi treatment shows a diminished effect on Distal-EJ in these cells, compared to WT cells. Shown are the mean Distal-EJ frequencies for WT and *Nbs1^n/h^* ES cells, each with an integrated EJ5-GFP reporter, which were transfected and treated with DMSO or ATMi, as described in Figure 2 (N = 6, error bars denote s.d.). (*) statistical difference between DMSO versus ATMi treatments of the same cell line (p≤0.0053), (†) statistical difference between WT and *Nbs1^n/h^* cells of the same treatment (p<0.0001). B. Proximal-EJ is not affected by Nbs1-deficiency. Shown are the mean frequencies of I-SceI-resistant p1, p2 amplification products (Proximal-EJ) for the transfection experiments described in A (N = 6, error bars denote s.d.). (*) as in A, p<0.0001 for WT, p = 0.0156 for *Nbs1^n/h^*. C. Distal End Utilization is elevated in Nbs1-deficient cells, and ATMi treatment shows a diminished effect on Distal End Utilization in these cells, compared to WT cells. Shown are the mean Distal End Utilization values of individual samples relative to DMSO-treated WT cells (N = 6, error bars denote s.d.). (*) as in A (p≤0.0011), (†) as in A (p≤0.0053).

As Nbs1 is important for activation of ATM following DSBs [6], [7], we considered that this factor might also affect end utilization. To test this hypothesis, we used an Nbs1-hypomorphic mouse ES cell line (*Nbs1^n/h^*), in which both alleles of the *Nbs1* gene are targeted [37], causing a 5-fold decrease in the level of Nbs1 protein [27]. The *Nbs1^n/h^* cell line containing EJ5-GFP was described previously, and shown to exhibit an elevated level of Distal-EJ, compared to WT cells [27]. To determine the role of Nbs1 on the relative efficiency of Proximal-EJ and Distal-EJ, as well as the effect of ATMi treatment on these EJ events, we performed the aforementioned I-SceI/Trex2 experiment using the *Nbs1^n/h^*-EJ5-GFP cell line. We found that *Nbs1^n/h^* cells exhibited a substantial increase in Distal-EJ repair relative to WT cells (5.1-fold, p<0.0001, Figure 4A). ATMi treatment of the *Nbs1^n/h^* cells caused a modest increase in Distal-EJ (1.4-fold, p = 0.0053, Figure 4A). Proximal-EJ was equally efficient in WT and *Nbs1^n/h^* cells, and ATMi treatment of the *Nbs1^n/h^* cells casued a slight reduction in the frequency of Proximal-EJ (1.3-fold, p = 0.0156, Figure 4B). Lastly, Distal End Utilization in *Nbs1^n/h^* cells was higher than in WT cells (4.9-fold, p<0.0001, Figure 4C), and was enhanced by ATMi treatment (1.8-fold, p = 0.0011, Figure 4C). Notably, the effect of ATMi on Distal End Utilization in *Nbs1^n/h^* cells (1.8-fold, Figure 4C) is substantially reduced as compared to the effect in WT cells (5.3-fold, Figure 2D, Figure 4C). In summary, these data indicate that Nbs1 is important to limit Distal End Utilization to a similar degree as ATM.

### The role of individual factors during Distal-EJ is not predictive of their effect on end processing

Apart from suppressing Distal End Utilization, we considered whether ATM and/or Nbs1 might also affect the degree of end processing during EJ. For this, we cloned Distal-EJ amplification products from GFP+ sorted cells following I-SceI and Trex2 co-expression of WT (DMSO and ATMi treated), *ATM^−/−^*, and *Nbs1^n/h^* cells (DMSO and ATMi treated), (p3, p2 products shown in Figure 2C, Figure S1B). For each condition, 30 independent clones were sequenced to determine the Distal-EJ repair junctions.

**Figure 5:**
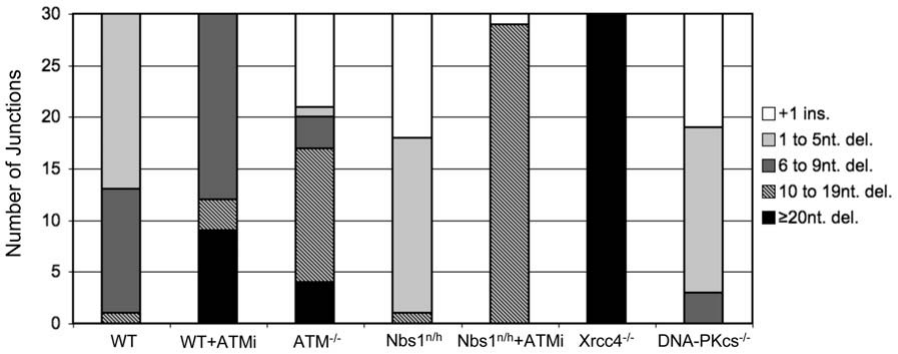
Discrete roles of individual factors on deletion size during Distal-EJ. ATM and Xrcc4 suppress longer deletions, whereas Nbs1 and DNA-PKcs promote short deletions. Distal-EJ products were amplified and cloned for sequencing analysis from GFP+ sorted samples following co-expression of I-SceI and Trex2. Shown is a summary of sequence analysis for WT cells, WT cells treated with ATMi, *ATM^−/−^* cells, *Nbs1^n/h^* cells, *Nbs1^n/h^* cells treated with ATMi, *DNA-PKcs^−/−^* cells, and *Xrcc4^−/−^* cells. Shown are the numbers of products (out of 30 total, sequences in Table S1) in five different classifications: +1 insertion (ins.), 1 to 5 nt. deletions (del.), 6 to 9 nt. del., 10 to 19 nt. del., and ≥20 nt. del.

As compared to an I-SceI+ Distal-EJ product, we classified the sequences into five groups: +1 insertion, 1 to 5 nt. deletions, 6 to 9 nt. deletions, 10 to 19 nt. deletions, and ≥20 nt. deletions (Figure 5 and Table S1). For WT cells, we found that Distal-EJ products showed mostly deletions of the I-SceI overhang region (17/30 with 1 to 5 nt. deletions), and the remaining clones showed only slightly larger deletions (12/30 with 6 to 9 nt. deletions, 1/30 with a 10 nt. deletion). For both ATMi treated WT cells and *ATM^−/−^* cells, we found an increase in the frequency of deletions greater than 9 nt., as compared to DMSO treated WT cells (12/30 for WT+ATMi, p = 0.0011; 17/30 for *ATM^−/−^*, p<0.0001; compared to 1/30 for WT). In contrast, for *Nbs1^n/h^* cells we found a reduction in clones showing deletions greater than 6 nt., in comparison to WT cells (1/30 for *Nbs1^n/h^* cells, p = 0.0004; compared to 13/30 for WT). In summary, while ATM and Nbs1 both suppress Distal End Utilization, these factors appear to show divergent effects on end processing, with ATM suppressing longer deletions, and Nbs1 promoting short deletions. Notably, for ATMi treated *Nbs1^n/h^* cells we found an increase in clones showing deletions greater than 9 nts., as compared to either WT or *Nbs1^n/h^* cells (29/30 for *Nbs1^n/h^* with ATMi, p<0.0001; compared to 1/30 for WT and 1/30 for *Nbs1^n/h^*). This latter result indicates that the increase in longer deletions caused by ATMi is dominant over the decrease in short deletions caused by the *Nbs1^n/h^* alleles.

The finding that Distal-EJ events in ATMi-treated cells show longer deletions, yet are promoted by c-NHEJ factors, indicates that deletion size may not necessarily be predictive of the involvement of the c-NHEJ pathway. This result is consistent with previous studies showing that while Xrcc4-deficiency causes longer deletion EJ products, DNA-PKcs-deficiency does not lead to elevated deletion sizes [38]–[41]. To confirm this distinction in our experiments, we performed the above sequence analysis using the *DNA-PKcs^−/−^* and *Xrcc4^−/−^* cell lines. Namely, we cloned Distal-EJ amplification products from GFP+ sorted cells following co-expression of I-SceI and Trex2 in these cell lines (p2, p3 products shown in Figure S1B), and subsequently sequenced 30 clones each (Figure 5, Table S1).

From this analysis, we found that junctions from *DNA-PKcs^−/−^* cells showed fewer deletions greater than 6 nts. in comparison to WT cells (3/30 for *DNA-PKcs^−/−^*, p = 0.0074; compared to 13/30 for WT), along with a number of 1–5 nt. deletions (16/30). Thus, *DNA-PKcs^−/−^* cells showed a shift towards shorter deletions as compared to WT cells, similar to the findings of *Nbs1^n/h^* cells. The rest of the *DNA-PKcs^−/−^* junctions were +1 insertions (11/30), which were not observed in WT cells, but were found in *ATM^−/−^* (9/30) and *Nbs1^n/h^* cells (12/30). In contrast, *Xrcc4^−/−^* cells show much more extensive deletions, with all clones showing ≥20 nt. deletions (30/30), compared to none with WT cells (p<0.0001). These results indicate that Xrcc4 is important to limit extensive deletions during Distal-EJ, whereas DNA-PKcs promotes short deletions.

Apart from variations in deletion size, use of microhomology and templated nucleotides are distinct between individual repair events, but these characteristics also are not necessarily predictive of the involvement of c-NHEJ. For instance, only clones from *Xrcc4^−/−^* cells showed any evidence of microhomology greater than 4 nt. (19/30 show a junction with 6 nt. of microhomology). The rest of the repair junctions observed in our experiments showed 0–4 nt. of microhomology, without any clear distinction between the cell lines. As the mechanistic requirements for limited microhomology during different EJ pathways is still unclear [42], [43], EJ events with 0–4 nts. of microhomology could be mediated by c-NHEJ factors or alt-EJ. Similarly, the +1 insertion events likely involve Family X DNA Polymerases (Pol X), which also could function during c-NHEJ or alt-EJ events [44]. Though, in this case, we observe an increase in +1 insertion events in cells deficient for Nbs1, ATM, and DNA-PKcs, which could reflect an improved recruitment of Pol X polymerases during EJ.

To summarize the junction analysis, we found that ATM and Xrcc4 limit the length of deletions, whereas Nbs1 and DNA-PKcs promote short deletions. In contrast, we found that Distal-EJ is suppressed via ATM and Nbs1, and that the elevated level of Distal-EJ caused by ATM-disruption requires both Xrcc4 and DNA-PKcs. These findings indicate that the role of individual factors during end processing is not predictive of their role during end utilization.

## Discussion

Limiting the use of incorrect ends during EJ repair of multiple chromosome breaks is likely an important aspect of genome maintenance, and hence tumor suppression. Using a method for quantifying end utilization during repair of two tandem DSBs, we present evidence that ATM and Nbs1 are important to limit Distal End Utilization. We also present evidence that the increase in Distal-EJ that is caused by ATM-disruption is dependent on c-NHEJ factors (DNA-PKcs, Xrcc4, and XLF). We suggest that ATM and Nbs1 may suppress genome rearrangements not only through activating the DDR, but also via promoting faithful end utilization during c-NHEJ. This notion is consistent with important previous studies showing that ATM supports correct utilization of hairpin coding ends during V(D)J recombination via c-NHEJ factors [13], [16]–[20]. Our findings indicate that such a role for ATM is not limited to Artemis-dependent c-NHEJ of hairpin ends generated by the Rag1/2 endonuclease, but is also important for Artemis-independent c-NHEJ repair of multiple DSBs with open ends. In summary, we suggest that cells that are deficient in ATM or Nbs1 are more prone to chromosome rearrangements during c-NHEJ of multiple DSBs.

In addition, we find that c-NHEJ-deficiency does not cause a substantial effect on Distal-EJ levels in ATM-proficient cells (within 2-fold of WT, Figure 3A). This finding is consistent with other studies showing that neither Ku70 nor Xrcc4 are required for chromosomal translocations that result from repair of multiple I-SceI-induced DSBs [45]–[48]. These studies have raised the possibility that c-NHEJ factors may not play a role in promoting chromosome rearrangements outside the programmed rearrangements during lymphocyte development. Rather, these studies suggested that alt-EJ mechanisms might be responsible for such chromosome rearrangements. However, we have presented evidence that c-NHEJ factors (Xrcc4, DNA-PKcs, and XLF) can promote genome rearrangements caused by ATM-deficiency. Thus, we suggest that c-NHEJ may indeed play a role during genome rearrangements, but specifically under conditions that enable incorrect end utilization (e.g. deficient in ATM or Nbs1).

### Break persistence versus end tethering

**Figure 6:**
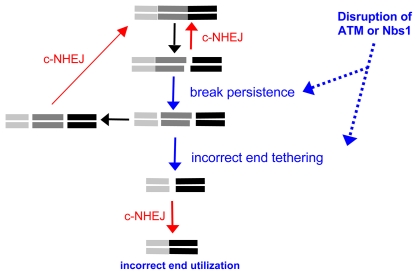
ATM limits incorrect end utilization during c-NHEJ of two tandem DSBs. Shown is a diagram of EJ repair of two tandem non-cohesive DSBs. Incorrect end utilization is shown to be caused by elevated break persistence and/or incorrect end tethering. Notably, even when individual breaks are more persistent, incorrect end tethering is still essential to generate the Distal-EJ product. ATM/Nbs1-deficiency is modeled to cause elevated break persistence and/or incorrect end tethering, leading to incorrect end utilization during c-NHEJ.

The increase in Distal-EJ versus Proximal-EJ caused by disruption of ATM (and/or Nbs1) could be due to at least two mechanisms: increased break persistence and/or defective end tethering (Figure 6). Considering the former, ATM-disruption could enhance the persistence of each DSB, thereby increasing the probability of both DSBs existing simultaneously, leading to more Distal End Utilization. This model is supported by findings that DSBs formed during V(D)J recombination in ATM-deficient cells persist longer, even through multiple cell doublings [12]–[15]. However, c-NHEJ-deficiency also causes an increase in break persistence [49], but does not lead to a substantial increase in Distal-EJ. Thus, not all conditions that lead to elevated break persistence appear to cause an increase in Distal-EJ. To summarize the break persistence model, ATM (and/or Nbs1) could be important to limit the persistence of DSBs, and thereby reduce the probability that multiple DSBs occur simultaneously, which would limit the frequency of chromosome rearrangements.

Alternatively, disruption of ATM and/or Nbs1 could cause defective end tethering, thereby increasing the probability of distal end synapsis, and hence Distal End Utilization. A role for Nbs1 during end synapsis is consistent with the DNA tethering activity of the Mre11-complex (Mre11-Rad50-Nbs1) [50]–[52]. Such tethering could be important not only for recruitment of the sister chromatid during homologous recombination, but also for end synapsis during EJ. ATM could support this tethering function of the Mre11-complex, as Nbs1 is a target of ATM kinase activity [6], [7]. Alternatively, since ATM is important for recruitment of a number of factors to chromatin to activate the DDR, such factors could stabilize damaged chromatin [53], and thereby support faithful end tethering during repair. In a related model, ATM could regulate the end tethering functions of c-NHEJ factors, since ATM can phosphorylate XLF [54] and DNA-PKcs [55], the latter of which can tether DNA molecules *in vitro* [56]. A role for ATM during these events is supported by findings that combined loss of *ATM* with *DNA-PKcs* or *Lig4* caused substantially elevated levels of broken mitotic chromosomes, as compared to either single mutant [15], [57]. Nbs1 could also be important for such ATM-dependent mechanisms of end tethering, since Nbs1 activates ATM kinase activity following DSBs [7]. Thus, ATM and/or Nbs1 could support the end tethering functions of either the Mre11 complex and/or c-NHEJ factors themselves, and thereby limit incorrect end utilization during EJ.

Of course, these two aspects of repair need not be mutually exclusive, as defects in end tethering could delay EJ causing increased break persistence, and vice versa. However, we suggest that even in situations of elevated DSB persistence, incorrect end tethering is still essential for generation of Distal-EJ products. In summary, we suggest that disruption of ATM (and/or Nbs1) leads to defective end tethering and/or elevated break persistence in a manner that results in a substantial elevation of incorrect end utilization during c-NHEJ repair of multiple DSBs (Figure 6).

### End utilization and end processing appear to be distinct processes

We also find that individual factors show distinct effects on end processing during EJ. The end processing observed in these experiments could be influenced by 5′ to 3′ end resection, and/or other mechanisms of DSB end degradation. Since Nbs1 appears to promote 5′ to 3′ end resection during *in vitro* EJ assays [58], this mechanism likely contributes to its role in promoting short deletions during EJ.

In contrast to Nbs1, we find that ATM appears to suppress deletions, which is supported by recent findings that ATM limits terminal end processing of DNA ends *in vitro*, and during plasmid EJ *in vivo* [59], [60]. Furthermore, we find that ATMi causes longer deletions even in the Nbs1-deficient cells. This result indicates that loss of ATM-mediated end protection may enable the low level of Nbs1 in these cells (5-fold reduced relative to WT [27]) to facilitate end resection. Alternatively, loss of ATM-mediated end protection could lead to an Nbs1-independent mechanism of end degradation. The former model is supported by a recent study showing that Mre11 promotes the terminal end processing caused by ATM-disruption [60]. Somewhat paradoxical to these findings of ATM-mediated end protection, ATM has been shown to promote end resection as measured by recruitment of ssDNA binding protein (RPA) to DSBs [61], although apparently not in all circumstances [62]. Perhaps ATM may limit terminal end resection, but promote extensive end resection [61], [63]. Such a model is consistent with studies in yeast that support a two-step end resection process [64].

Notably, ATM and Nbs1 affect end processing in different directions, while both suppress Distal End Utilization. We also find a distinction between Xrcc4 versus DNA-PKcs. Namely, we find that Xrcc4 is important to limit the extent of deletions during EJ, while DNA-PKcs promotes short deletions. This distinction is consistent with other reports [38]–[41], as well as the notion that c-NHEJ is a modular and flexible process that can result in a variety of products [42]. In contrast, we find that both Xrcc4 and DNA-PKcs are important for the elevated level of Distal-EJ caused by ATM-disruption. In summary, these studies of ATM, Nbs1, Xrcc4, and DNA-PKcs indicate that the regulation of end processing appears to be distinct from that of end utilization.

### Therapeutic relevance

In conclusion, correct end utilization is likely an important mechanism for limiting chromosome rearrangements that can lead to cancer development. While disruption of ATM kinase activity may be beneficial for promoting tumor cell death via clastogenic agents [31], [65], such a therapeutic strategy may also disrupt faithful end utilization in non-tumor cells, which could lead to therapy-related malignancies. Conversely, developing therapeutic strategies to enhance faithful end utilization in non-tumor cells could have the potential to reduce therapy-related malignancies. As well, since meganucleases are being developed as potential genome engineering tools [66], we suggest that Trex2 expression could enhance mutagenesis around the DSB site of meganucleases. However, as such nucleases may form DSBs at multiple sites, we also suggest that functional ATM would be critical for limiting genome rearrangements during such a therapeutic approach.

## Materials and Methods

### Cell lines

*XLF^−/−^* [34], *DNA-PKcs^−/−^* [32], and *Artemis^−/−^* [36] ES cells were generously provided by Dr. Frederick Alt, and *ATM^−/−^* ES cells [30] were generously provided by Dr. Yang Xu. Cells (10^7^ in 0.8 ml Optimem, Invitrogen) were electroporated with 70µg of XhoI digested pim-EJ5-GFP at 710–720V/10µF. Hygromycin B selection (0.12 mg/ml) was used to select for targeting to the *pim1* locus in *XLF^−/−^*, *DNA-PKcs^−/−^* and *Artemis^−/−^* cells, as confirmed by PCR analysis [28]. Puromycin selection (1.2 µg/ml) was used to select random integrants of EJ5-GFP in *ATM^−/−^* cells. Integration of an intact copy of the reporter in *ATM^−/−^* cells was confirmed by Southern blot analysis, as described previously [28]. Other cell lines with chromosomally integrated EJ5-GFP were described previously: WT ES (AB2.2), *Nbs1^n/h^* ES, *Xrcc4^−/−^* ES, and HEK293 [27], [28].

### Repair assays

Mouse ES and HEK293 cells were cultured as described previously [28], and 10^5^ cells were plated the day before an incubation with a mixture of 0.8µg of pCBASce, 0.4µg of pCAGGS-Trex2, and 3.6µL of Lipofectamine 2000 (Invitrogen), in 1ml of antibiotic-free media [27]. After 3 hr, the transfection media was removed and replaced with complete media containing either 10µM ATMi [31](EMD Biosciences) or DMSO (vehicle). Subsequently (3 days), half of each transfection sample was analyzed by FACS (CyAN ADP, Dako) to determine %GFP+ cells (Distal-EJ), and the other half was used to isolate genomic DNA for determination of Proximal-EJ, as described previously [27]. Briefly, genomic DNA was amplified using EJ5purF (p1, 5′ agcggatcgaaattgatgat) and KNDRR (p2, 5′ aagtcgtgctgcttcatgtg). The amplification products were purified (GFX, GE), and digested with I-SceI (NEB), separated on agarose gels, and detected with ethidium bromide, where complete digestion was confirmed with parallel samples from untransfected cells. The percentage of I-SceI-resistant product was calculated from the relative staining intensity of I-SceI+ versus I-SceI-resistant bands within the same lane, as described previously [27], [29].

For single clone analysis, we performed the same transfection protocol, except we included 0.4µg of dsRED-N1 (Clontech) and a total of 4.8µL of Lipofectamine 2000. Three days after transfection, we enriched for transfected cells by sorting dsRED+ cells, which we plated at low density to isolate single clones. For each clone, we determined whether the 5′ and 3′ I-SceI sites had been disrupted, using the Proximal-EJ assay described above, where the 5′ I-SceI site was analyzed using the primers KNDRF (p3, 5′ ctgctaaccatgttcatgcc) and EJ5purR (p4, 5′ cttttgaagcgtgcagaatg) [27].

To calculate Distal End Utilization for individual samples, the percentage of GFP+ cells was divided by the percentage of I-SceI-resistant amplification products. To facilitate comparison to WT, each individual Distal End Utilization value was divided by the mean value for WT DMSO treated cells. We amplified Distal-EJ products from GFP+ sorted cells from representative transfections, using KNDRF (p3) and KNDRR (p2). The amplification products were digested with I-SceI, as above. I-SceI-resistant bands were isolated and cloned into TA vectors (Invitrogen) for sequencing with the M13R primer.

### Statistical analysis

For comparison of EJ frequencies, we used Student's unpaired *t*-test. For comparison of Distal-EJ breakpoint junctions, we used Fisher's Exact Test.

## Supporting Information

Figure S1 Details of EJ assays. A. The Proximal-EJ assay is quantitative within two-fold. WT mouse ES cells were transfected with an expression vector for I-SceI (S), along with either the Trex2 expression vector (S+Trex2), or empty vector (S+EV). Following transfection, genomic DNA was isolated from S+Trex2 cells, and also from an equal mixture of S+Trex2 cells and S+EV cells. As I-SceI-resistant Proximal-EJ products require Trex2 expression (see Figure 2B), the mixed sample should show a 2-fold reduction in such products, as compared to the S+Trex2 sample. Shown are representative Proximal-EJ products (left) along with the mean Proximal-EJ value from separate transfections used to generate independent samples (right, N = 3, error bars denote s.d.). (*) statistical difference between S+Trex2 versus the equal mixture of S+EV and S+Trex2, p = 0.0009. Also shown (left) are Proximal-EJ products of an S+Trex2 transfection of *Xrcc4^−/−^* cells, performed in parallel. B. Trex2 and I-SceI co-expression leads to Distal-EJ products that are I-SceI-resistant. Several cell types with the EJ5-GFP reporter (WT ES treated with DMSO or ATMi, *ATM^−/−^*, *Nbs1^n/h^*, *Xrcc4^−/−^*, *DNA-PKcs^−/−^*, and *XLF^−/−^*) were transfected as in A. Subsequently, GFP+ Distal-EJ products were sorted and the restoration of the I-SceI site was determined by PCR amplification and I-SceI digestion analysis as in Figure 2C. Shown are uncut (U) and I-SceI-digested (S) products from these samples. Some of these products were also shown in Figure 2C, which we show here to enable comparison. C. Formation of I-SceI-resistant Proximal-EJ products is dependent on Trex2 expression, including in *ATM^−/−^* cells. Shown are representative Proximal-EJ samples from S+EV and S+Trex2 transfection of *ATM^−/−^* cells, as described in A. D. ATMi treatment causes an increase in Distal End Utilization when the end-point analysis is performed at either 3 or 6 days. WT mouse ES cells were transfected as in A, and cultured for 3 or 6 days prior to determining Distal End Utilization values as described in Figure 2D. Shown are the mean Distal End Utilization values for independent transfections for 3 and 6 days end points (N≥3, error bars denote s.d.). (*) distinct from DMSO treatment from the same end point, p<0.0014; values were not statistically different between 3 and 6 days.
(0.81 MB PDF)

Figure S2 Efficiency of I-SceI-induced DSBs at tandem recognition sites; ATM limits Distal End Utilization in HEK293 cells. A. ATM does not inhibit formation of I-SceI-induced DSBs at both tandem I-SceI sites. WT mouse ES cells were transfected with expression plasmids for I-SceI, Trex2, and dsRED. Also, transfections were treated with DMSO or ATMi as in Figure 2. Following transfection (3 days), dsRED+ cells were sorted to enrich for transfected cells, and were plated at low density to isolate single clones. Loss of the 5′ and 3′ I-SceI-recognition sites was determined by PCR amplification and I-SceI digestion for individual clones, using the primers depicted in the diagram. Shown (left) are representative clones with loss of both the 5′ and 3′ I-SceI sites (Clone 1), loss of only the 3′ site (Clone 2), and loss of only the 5′ site (Clone 3). Also shown (right) are the percentages of clones that have lost one I-SceI site (5′ or 3′ S-, e.g. Clones 3 or 2, respectively) versus both sites (5′ and 3′ S-, e.g. Clone 1), for DMSO and ATMi treated samples. B. ATM suppresses incorrect end utilization in HEK293 cells. HEK293 cells with an integrated copy of EJ5-GFP were co-transfected with expression plasmids for I-SceI and Trex2 and treated with ATMi or DMSO. Shown are the mean frequencies of Distal-EJ (left), Proximal-EJ (middle), and Distal End Utilization (right) for these samples, determined as in Figure 2 (N = 6, error bars denote s.d.). (*) statistical difference between DMSO and ATMi treatment (p<0.0001).
(0.35 MB PDF)

Table S1 Sequences of Distal-EJ junctions. For reference, shown is the unmodified I-SceI site in capital letters with the cleavage site marked by a slash, which would be generated by Distal-EJ that restores the I-SceI site. Shown are the five categories of products shown in Figure 5, along with the sequences of each individual repair product. Inserted nucleotides are in bold, substituted nucleotides are in italics and bold, and microhomology is underlined. Shown are the numbers of each product, out of 30 total, from analysis of Distal-EJ products (GFP+ cells), following co-expression of I-SceI and Trex2, from a number of cell types: WT ES treated with DMSO, WT ES treated with ATMi, *ATM^−/−^*, *Nbs1^n/h^*, *Nbs1^n/h^* treated with ATMi, *Xrcc4^−/−^*, and *DNA-PKcs^−/−^* (the p3, p2 I-SceI-resistant amplification products are shown in Figure 2C, Figure S1B).
(0.08 MB PDF)
