# Supplementary figures and images for: ATM Limits Incorrect End Utilization during Non-Homologous End Joining of Multiple Chromosome Breaks
Source: PLoS Genet. 2010 Nov 4;6(11):e1001194. doi: 10.1371/journal.pgen.1001194 (PMC2973825; doi:10.1371/journal.pgen.1001194)

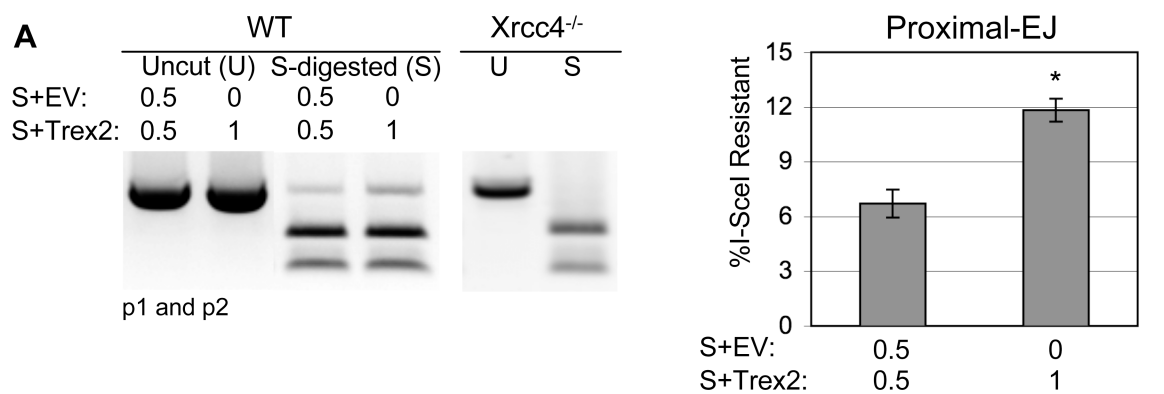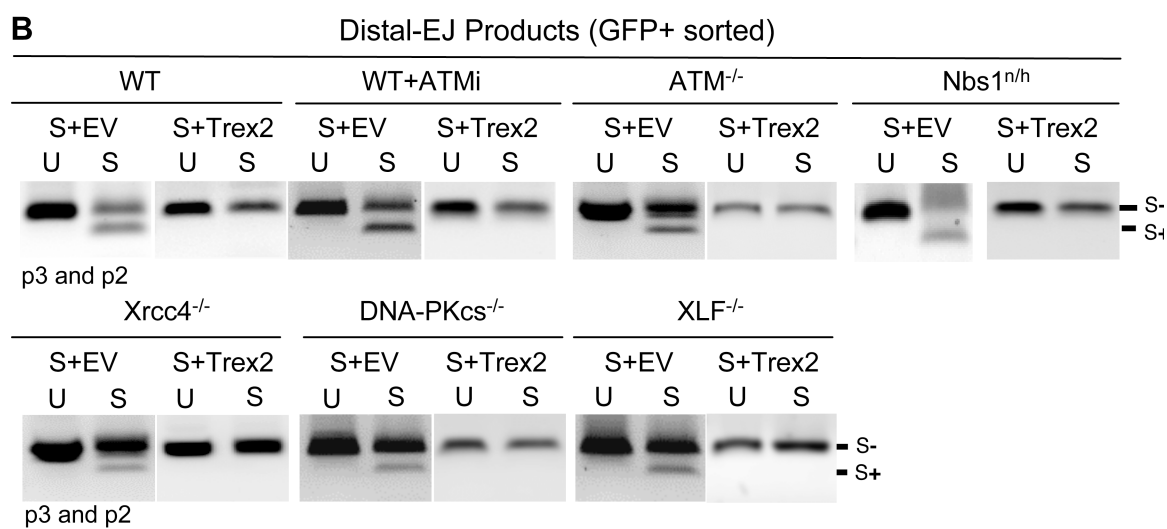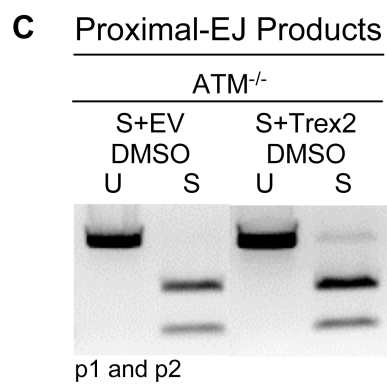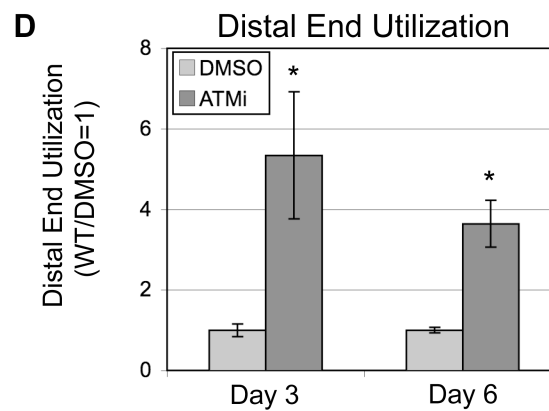

Supplement: Figure S1 — Details of EJ assays. A. The Proximal-EJ assay is quantitative within two-fold. WT mouse ES cells were transfected with an expression vector for I-SceI (S), along with either the Trex2 expression vector (S+Trex2), or empty vector (S+EV). Following transfection, genomic DNA was isolated from S+Trex2 cells, and also from an equal mixture of S+Trex2 cells and S+EV cells. As I-SceI-resistant Proximal-EJ products require Trex2 expression (see Figure 2B), the mixed sample should show a 2-fold reduction in such products, as compared to the S+Trex2 sample. Shown are representative Proximal-EJ products (left) along with the mean Proximal-EJ value from separate transfections used to generate independent samples (right, N = 3, error bars denote s.d.). (*) statistical difference between S+Trex2 versus the equal mixture of S+EV and S+Trex2, p = 0.0009. Also shown (left) are Proximal-EJ products of an S+Trex2 transfection of Xrcc4−/− cells, performed in parallel. B. Trex2 and I-SceI co-expression leads to Distal-EJ products that are I-SceI-resistant. Several cell types with the EJ5-GFP reporter (WT ES treated with DMSO or ATMi, ATM−/−, Nbs1n/h, Xrcc4−/−, DNA-PKcs−/−, and XLF−/−) were transfected as in A. Subsequently, GFP+ Distal-EJ products were sorted and the restoration of the I-SceI site was determined by PCR amplification and I-SceI digestion analysis as in Figure 2C. Shown are uncut (U) and I-SceI-digested (S) products from these samples. Some of these products were also shown in Figure 2C, which we show here to enable comparison. C. Formation of I-SceI-resistant Proximal-EJ products is dependent on Trex2 expression, including in ATM−/− cells. Shown are representative Proximal-EJ samples from S+EV and S+Trex2 transfection of ATM−/− cells, as described in A. D. ATMi treatment causes an increase in Distal End Utilization when the end-point analysis is performed at either 3 or 6 days. WT mouse ES cells were transfected as in A, and cultured for 3 or 6 days prior to determining [file pgen.1001194.s001.pdf]

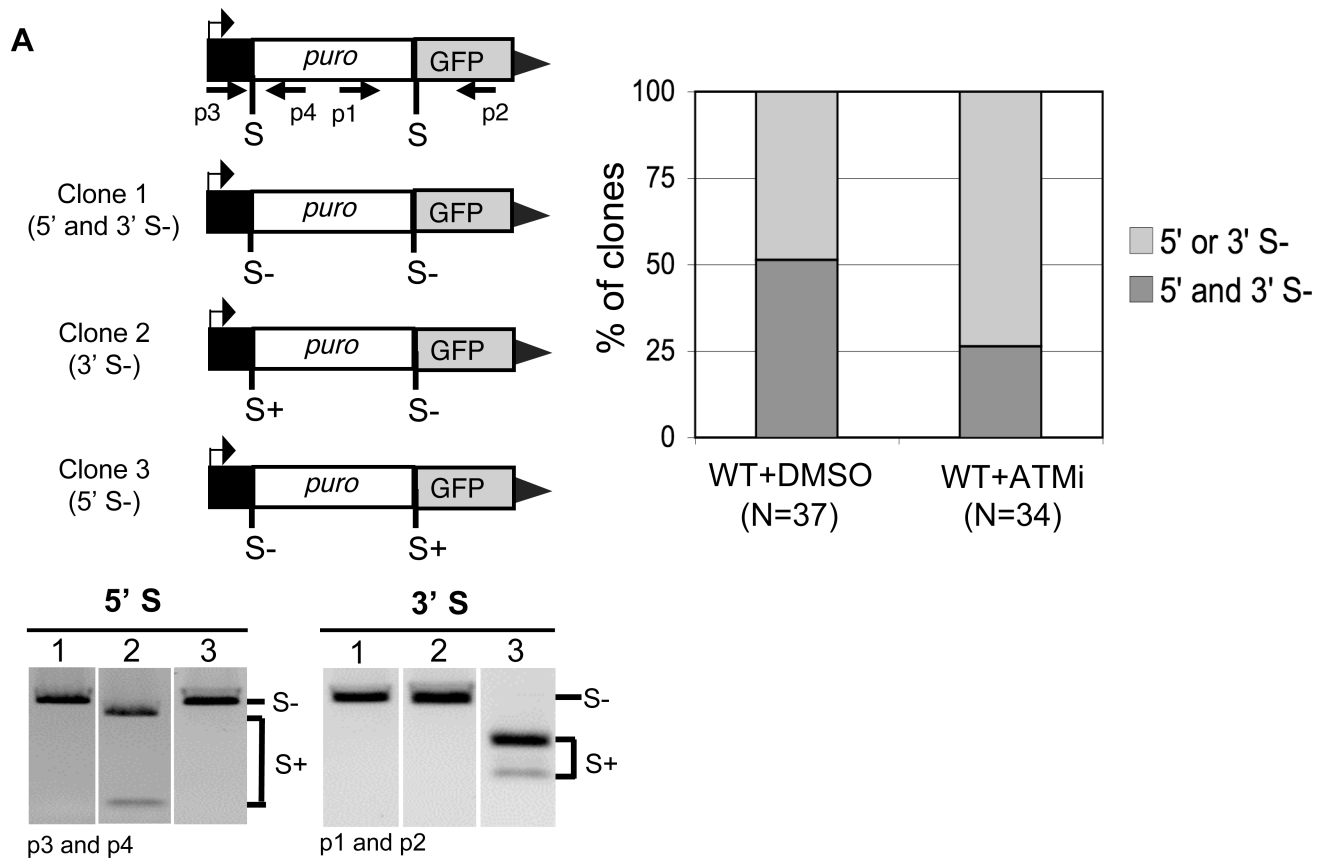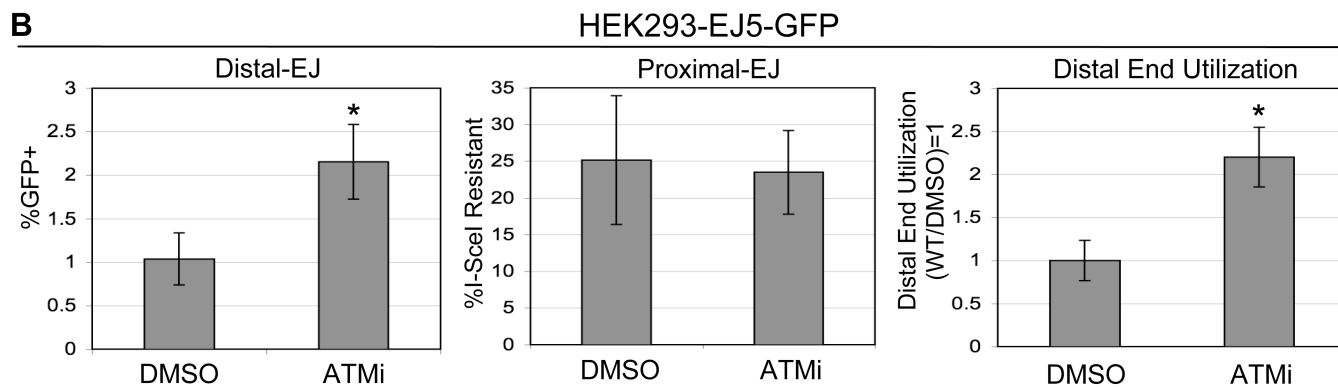

Supplement: Figure S2 — Efficiency of I-SceI-induced DSBs at tandem recognition sites; ATM limits Distal End Utilization in HEK293 cells. A. ATM does not inhibit formation of I-SceI-induced DSBs at both tandem I-SceI sites. WT mouse ES cells were transfected with expression plasmids for I-SceI, Trex2, and dsRED. Also, transfections were treated with DMSO or ATMi as in Figure 2. Following transfection (3 days), dsRED+ cells were sorted to enrich for transfected cells, and were plated at low density to isolate single clones. Loss of the 5′ and 3′ I-SceI-recognition sites was determined by PCR amplification and I-SceI digestion for individual clones, using the primers depicted in the diagram. Shown (left) are representative clones with loss of both the 5′ and 3′ I-SceI sites (Clone 1), loss of only the 3′ site (Clone 2), and loss of only the 5′ site (Clone 3). Also shown (right) are the percentages of clones that have lost one I-SceI site (5′ or 3′ S-, e.g. Clones 3 or 2, respectively) versus both sites (5′ and 3′ S-, e.g. Clone 1), for DMSO and ATMi treated samples. B. ATM suppresses incorrect end utilization in HEK293 cells. HEK293 cells with an integrated copy of EJ5-GFP were co-transfected with expression plasmids for I-SceI and Trex2 and treated with ATMi or DMSO. Shown are the mean frequencies of Distal-EJ (left), Proximal-EJ (middle), and Distal End Utilization (right) for these samples, determined as in Figure 2 (N = 6, error bars denote s.d.). (*) statistical difference between DMSO and ATMi treatment (p<0.0001). (0.35 MB PDF) [file pgen.1001194.s002.pdf]
