# Supplementary material for: ATM Limits Incorrect End Utilization during Non-Homologous End Joining of Multiple Chromosome Breaks
Source: PLoS Genet. 2010 Nov 4;6(11):e1001194. doi: 10.1371/journal.pgen.1001194 (PMC2973825; doi:10.1371/journal.pgen.1001194)
Supplement: Table S1 — Sequences of Distal-EJ junctions. For reference, shown is the unmodified I-SceI site in capital letters with the cleavage site marked by a slash, which would be generated by Distal-EJ that restores the I-SceI site. Shown are the five categories of products shown in Figure 5, along with the sequences of each individual repair product. Inserted nucleotides are in bold, substituted nucleotides are in italics and bold, and microhomology is underlined. Shown are the numbers of each product, out of 30 total, from analysis of Distal-EJ products (GFP+ cells), following co-expression of I-SceI and Trex2, from a number of cell types: WT ES treated with DMSO, WT ES treated with ATMi, ATM−/−, Nbs1n/h, Nbs1n/h treated with ATMi, Xrcc4−/−, and DNA-PKcs−/− (the p3, p2 I-SceI-resistant amplification products are shown in Figure 2C, Figure S1B). (0.08 MB PDF) [file pgen.1001194.s003.pdf]

**TABLE S1. Breakpoint Junction Sequences**								
**I-SceI Site(CAPS)**<br>caaagaattcTAGGGATAA/CAGGGTAATggatccaccg	**micro-homology**	**WT**	**WT + ATMi**	**$ATM^{-/-}$**	**$Nbs1^{n/h}$**	**$Nbs1^{n/h}$ + ATMi**	**$Xrcc4^{-/-}$**	**DNA $PKcs^{-/-}$**
**insertions / deletions**								
**insertion+1**		**0/30**	**0/30**	**9/30**	**12/30**	**1/30**	**0/30**	**11/30**
TAGGGATAA**A**CAGGGTAAT. **1nt. insertion**	2			9	12	1		11
**deletion 1-5nt.**		**17/30**	**0/30**	**1/30**	**17/30**	**0/30**	**0/30**	**16/30**
TAGGG...CAGGGTAAT 4nt. deletion	0	16		1				
TAGG…CAGGGTAAT 5nt. deletion	0				2			
TAGGGA…GGGTAAT 5nt. deletion	1	1			15			16
**deletion 6-9nt.**		**12/30**	**18/30**	**3/30**	**0/30**	**0/30**	**0/30**	**3/30**
TAG…CAGGGTAAT 6nt. deletion	0		1					
TAGGG...GGTAAT 7nt. deletion	1		2					
TA…CAGGGTAAT 7nt. deletion	1		1					
TAGGG...TAAT 9nt. deletion	4	11	5	1				1
caaagaattc…CAGGGTAAT 9nt. deletion	0	1	8	1				2
TAGG…**A**TAAT 9nt. deletion, **1nt. insertion**	0		1					
TAGGG...T***G***AT 9nt. deletion, ***1nt. substitution (G)***	4			1				
**deletion 10-19nt.**		**1/30**	**3/30**	**13/30**	**1/30**	**29/30**	**0/30**	**0/30**
caaagaatt...CAGGGTAAT 10nt. deletion	1	1						
TAGGG…**T**AT 10nt. deletion, **1nt. insertion**	0		3					
caaa...ATAACAGGGTAAT 11nt. deletion	0				1			
caaaga...AGGGTAAT 14nt. deletion	1					25		
...ACAGGGTAAT 19nt. deletion	0			1				
CAGGGTAATggatccaccg 19nt. deletion	2			12		4		
**deletion ≥20nt.**		**0/30**	**9/30**	**4/30**	**0/30**	**0/30**	**30/30**	**0/30**
caaag...AATggatccaccg 20nt. deletion	3						6	
caaaga...atccaccg 24nt. deletion	2			4				
...atccaccg 37nt. deletion	2						2	
tcctgggc…AACAGGGTAAT 52nt. deletion	3		9					
gtgctg…tctcatcattttggcaaaga…(I-SceI site)…atggtgagca 12nt. del., 41nt. del.	6,2						19	
ggttattgtg…a***g***catggtga 61nt. deletion***, 1nt. substitution (g)***	0						1	
cagctcctgg…ccaccatggt 79nt. deletion	1						2	
